# Horizontal gene transfer from Bacteria to rumen Ciliates indicates adaptation to their anaerobic, carbohydrates-rich environment

**DOI:** 10.1186/1471-2164-7-22

**Published:** 2006-02-10

**Authors:** Guénola Ricard, Neil R McEwan, Bas E Dutilh, Jean-Pierre Jouany, Didier Macheboeuf, Makoto Mitsumori, Freda M McIntosh, Tadeusz Michalowski, Takafumi Nagamine, Nancy Nelson, Charles J Newbold, Eli Nsabimana, Akio Takenaka, Nadine A Thomas, Kazunari Ushida, Johannes HP Hackstein, Martijn A Huynen

**Affiliations:** 1 Center for Molecular and Biomolecular Informatics, Nijmegen Center for Molecular Life Sciences, Radboud University Nijmegen Medical Centre, Toernooiveld 1, 6525 ED Nijmegen, The Netherlands; 2 Institute of Rural Sciences, University of Wales, Aberystwyth, SY23 3AL, UK; 3 I.N.R.A., Station de Recherches sur la Nutrition des Herbivores, Centre de Recherches de Clermont-Ferrand/Theix, France; 4 National Institute of Livestock and Grassland Science, 2 Ikenodai, Kukizaki, Ibaraki, 305-0901, Japan; 5 Rowett Research Institute, Aberdeen, AB21 9SB, UK; 6 Kielanowski Institute of Animal Physiology and Nutrition, Polish Academy of Sciences, Jablonna, Warsaw, Poland; 7 Rumen Microbiology Research Team, STAFF-Institute, 446-1 Ippaizuka, Kamiyokoba, Tsukuba 305-0854, Japan; 8 Laboratory of Animal Science, Kyoto Prefectural University, Shimogamo, Kyoto 606-8522, Japan; 9 Department of Evolutionary Microbiology, Radboud University Nijmegen, Nijmegen, The Netherlands

## Abstract

**Background:**

The horizontal transfer of expressed genes from Bacteria into Ciliates which live in close contact with each other in the rumen (the foregut of ruminants) was studied using ciliate Expressed Sequence Tags (ESTs). More than 4000 ESTs were sequenced from representatives of the two major groups of rumen Cilates: the order Entodiniomorphida (*Entodinium simplex, Entodinium caudatum, Eudiplodinium maggii, Metadinium medium, Diploplastron affine, Polyplastron multivesiculatum *and *Epidinium ecaudatum*) and the order Vestibuliferida, previously called Holotricha (*Isotricha prostoma, Isotricha intestinalis *and *Dasytricha ruminantium*).

**Results:**

A comparison of the sequences with the completely sequenced genomes of Eukaryotes and Prokaryotes, followed by large-scale construction and analysis of phylogenies, identified 148 ciliate genes that specifically cluster with genes from the Bacteria and Archaea. The phylogenetic clustering with bacterial genes, coupled with the absence of close relatives of these genes in the Ciliate *Tetrahymena thermophila*, indicates that they have been acquired via Horizontal Gene Transfer (HGT) after the colonization of the gut by the rumen Ciliates.

**Conclusion:**

Among the HGT candidates, we found an over-representation (>75%) of genes involved in metabolism, specifically in the catabolism of complex carbohydrates, a rich food source in the rumen. We propose that the acquisition of these genes has greatly facilitated the Ciliates' colonization of the rumen providing evidence for the role of HGT in the adaptation to new niches.

## Background

Horizontal Gene Transfer (HGT) implicates the transfer of genetic material between species. Genes acquired by this process can provide novel functions to the recipient organism, particularly when that organism is naive for functions associated with the newly acquired gene(s). Therefore, HGT has the potential to play an important role in the exploitation of new niches. HGT has been inferred in many biological processes including the emergence and spread of virulence-factors, resistance to antibiotics, and the long-term maintenance of organelles [1]. Thus far, HGT on a large-scale has mainly been described from organelles to the nucleus transfer [2], and between different species of Bacteria and Archaea [3,4], and on a smaller scale from Eukaryotes to Bacteria [5]. The least well-documented form of large-scale HGT deals with the uptake of DNA into eukaryotic cells. Individual examples include transfer from Bacteria to Fungi [6] or Ciliates [7] in the rumen. The transfer of 16 bacterial genes to Nematodes [8] and of 96 such genes to *Entamoeba histolytica *[9] are the only examples where HGT from Bacteria to Eukaryotes has been investigated on a large-scale.

Here we investigate HGT from the Bacteria to rumen Ciliates – a monophyletic but rather diverse group of unicellular Eukaryotes. These organisms co-exist in the rumen under conditions that have been shown to allow HGT *in vitro *[10,11]. Ciliates form an extremely diverse taxonomic group of protozoa with an enormous diversity of known species. They are the most complex single cell Eukaryotes, some having genomes with more than 30,000 genes [12]. They are abundant in almost every aqueous environment, from ocean waters to small ponds and even pockets of soil water; and they can grow as symbionts, commensals or parasites in pelagic, benthic, sapropelic or intestinal ecosystems. One of the intestinal environments in which Ciliates have been described is the rumen, a highly specialized foregut differentiation in herbivorous mammals like cattle, sheep and goats. In these animals the rumen is the primary site for digestion of plant material consumed as a food source. Digestion is performed by a numerous and diverse microbiota including Bacteria, anaerobic Fungi, and Ciliates. The resulting fermentation products, such as short-chain fatty acids, but also the microbial biomass substantially contribute to the nutrition of the host. There is a certain degree of genomic plasticity within this environment, with some evidence for HGT between organisms [6] and one bacterial species being naturally transformable [11].

Given the close contact between Ciliates and Bacteria in the rumen, this environment promises optimal conditions to study HGT from Bacteria to Eukaryotes, in particular, because Ciliates engulf and digest Bacteria [13]. In the process of breakdown and digestion of the Bacteria, some of the bacterial DNA may be taken up by the Ciliates and incorporated into their genomes.

Thus far, there have been reports of HGT to the rumen Ciliates of a xylanase [7], a cellulase [14] and a glutamate dehydrogenase [15]. Whether these are incidental occurrences or whether there is indeed evidence for large-scale HGT from Bacteria to Ciliates within this environment remained to be discovered. Here we have undertaken random cDNA sequencing of rumen dwelling Ciliates in order to identify expressed HGT candidates. As part of the EU-funded programs ERCULE (European Rumen ciliates CULturE collection), and CIMES (CIliates as Monitors for the Environmental Safety of GMOs) cDNA libraries were constructed from ten species of rumen Ciliates and were sequenced randomly (these ciliates were cultivated as mono-cultures in fistulated sheep). Thus we have obtained a large set of cDNAs (4768 sequences) from Ciliates belonging to the order Entodiniomorphida: i.e. *Entodinium simplex, Entodinium caudatum, Eudiplodinium maggii, Metadinium medium, Diploplastron affine, Polyplastron multivesiculatum *and *Epidinium ecaudatum *and from *Isotricha prostoma, Isotricha intestinalis *and *Dasytricha ruminantium*, which belong to the order Vestibuliferida. Here we examine these EST data for HGT from Bacteria. Using large-scale sequence comparisons and phylogenetic analyses we find 148 genes that are likely to have been horizontally transferred to the Ciliates. The majority of these genes are involved in the catabolism of complex carbohydrates and in the adaptation to an anaerobic environment. This supports the hypothesis that HGT plays an important role in the exploitation of new niches.

## Results

### Best Hit within the Bacteria

**Figure 1:**
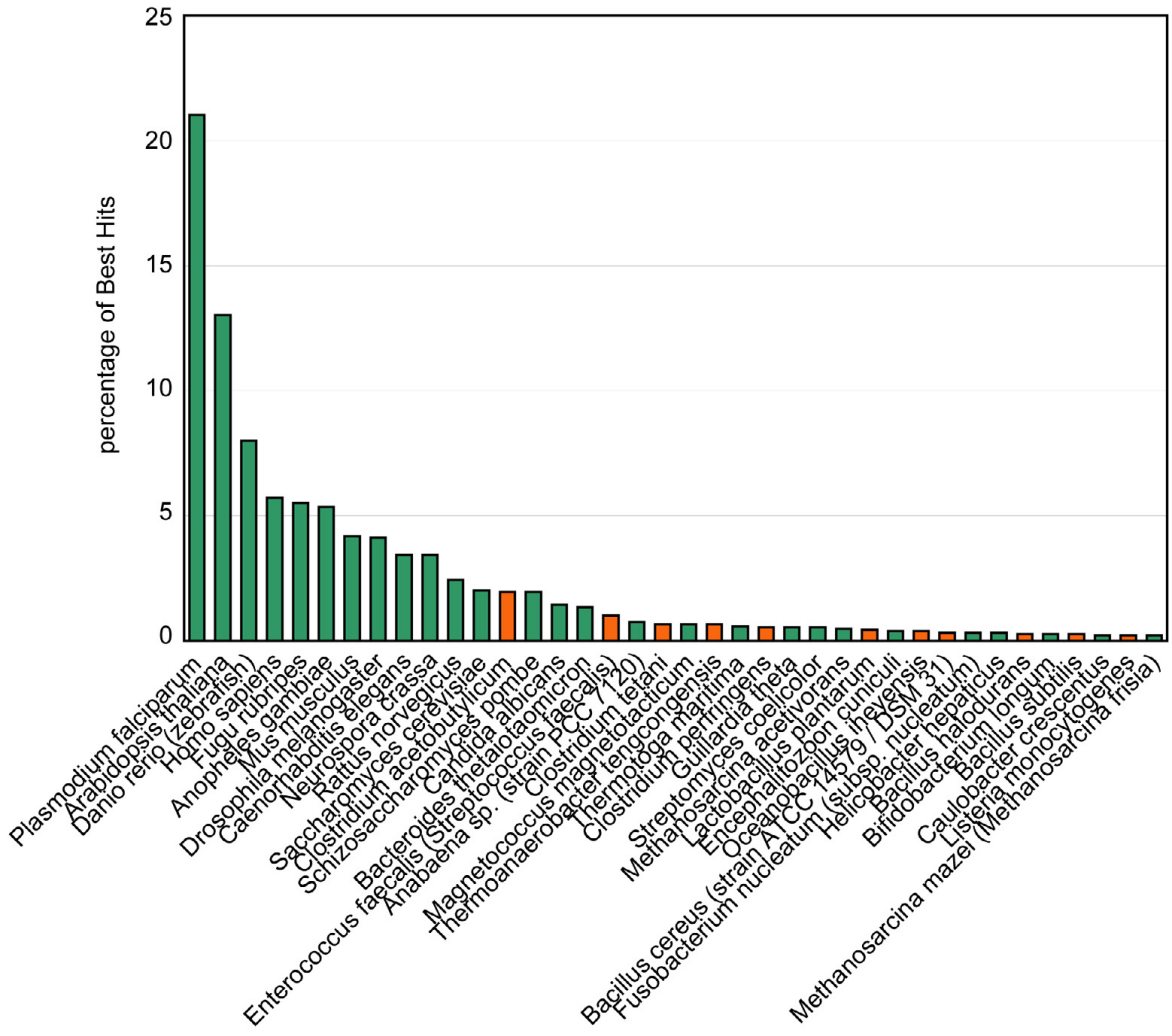
**Distribution of Best Hits over the proteomes**. Only proteomes with a number of Best Hits ≥ 0.2% are displayed. The 12 most represented proteomes are eukaryotic, the remaining set contains a large fraction of Firmicutes proteomes (orange).

A total of 4768 sequences were generated. Following filtering of sequences (see methods section) we clustered the remaining 4324 ESTs to remove redundancy. Grouping sequences with >97% identity over a stretch of at least 100 nucleotides resulted in 377 clusters containing between two and 21 sequences, and 3186 single sequence clusters (More details in Table 1). In order to find potential cases of HGT, we first performed a Best Hit search (translated nucleotides versus protein) against the predicted proteomes of 148 completely sequenced genomes. Examining the longest sequence per cluster resulted in 2307 clusters out of 3563 (64.75%) with a Best Hit in one of the complete genomes. The species distribution of the Best Hits (Figure 1), ranging from *Plasmodium falciparum *with the most hits, followed by *Arabidopsis thaliana *and *Danio rerio *is in agreement with the phylogeny of the Eukaryotes proposed by Baldauf *et al. *[16]. According to that phylogeny the sub-class Ciliophora is most closely related to the Apicomplexa (*P. falciparum*), then to the Viridiplantae (*A. thaliana*) and then to the Opisthokonts (*D. rerio, Homosapiens*, *Neurosporacrassa*).

Of the 38 Best Hit proteomes shown in Figure 1, the top 12 proteomes are eukaryotic. Nevertheless, we also found a substantial number of ESTs with a bacterial Best Hit. The Bacterium with the most Best Hits is *Clostridiumacetobutylicum*, a Firmicute which has previously been isolated from bovine rumen fluid [17]. A total of 11 different Firmicutes were identified with Best Hits, plus nine "other" Bacteria and two Archaea species.

Firmicutes, as with other "intestinal" Bacteria are likely HGT donors because they live in close contact with the studied Ciliates in the gastrointestinal tract of the ruminants [18]. According to Edwards *et al*., low G+C Gram positive Bacteria represent 54% of the rumen bacterial ecosystem, followed by the *Cytophaga-Flexibacter-Bacteroides *group (40%) [19]. Nelson *et al. *(2003) also show that Gram negative Bacteria were poorly represented in the gastrointestinal tract of wild herbivores [20].

A Best Hit approach can only provide an indication of the relationship between sequences in different organisms, and it does not always reflect the closest neighbour [21]. Therefore, we used a phylogenetic approach to further analyse those ciliate sequences which have a Best Hit in the bacterial genomes.

Furthermore we show details of the complete SWX comparison against the 148 proteomes in Additional file 2. Among 292 sequences with a Best Hit in Bacteria, 138 (47%) only hit Bacteria and this number raise to 151 (52%) that hit both Bacteria and Archaea.

### Phylogenetic analysis

Of the 362 sequences that have a bacterial sequence as Best Hit, 224 had enough homologs (minimally three) to construct phylogenetic trees (see Methods). In 133 of these 224 trees, the ciliate sequence clusters within the Bacteria (in these trees the second-smallest partition of the tree that contains the ciliate sequence and otherwise only contains Bacterial sequences, see Methods). Further examination of these 133 trees shows that in 34 trees the ciliate sequence clusters within Firmicutes, in nine trees within Proteobacteria, in three trees within Actinobacteria, in three trees within Bacteroidetes and in one tree within Spirochetes. In the remaining 83 trees the ciliate sequence clustered within a taxonomically more varied set of Bacteria. We also considered 13 ciliate sequences that clustered between the Bacteria and Archaea as HGT candidates, as well as two that clustered within the Archaea. Thus a total of 148 sequences were studied in more detail. We included all trees that showed evidence of HGT, irrespective of their statistical support, because we are interested in an estimate of the amount of HGT. The dominance of one functional class among the HGT candidates (see below) indicates the robustness of our results.

No bias of codon usage was detected, indicating complete adaptation to the codon usage of the Ciliate host and confirming that the HGT candidates are not contaminations (data not shown).

### Over-representation of genes involved in anaerobic metabolism among HGT candidates

**Figure 2:**
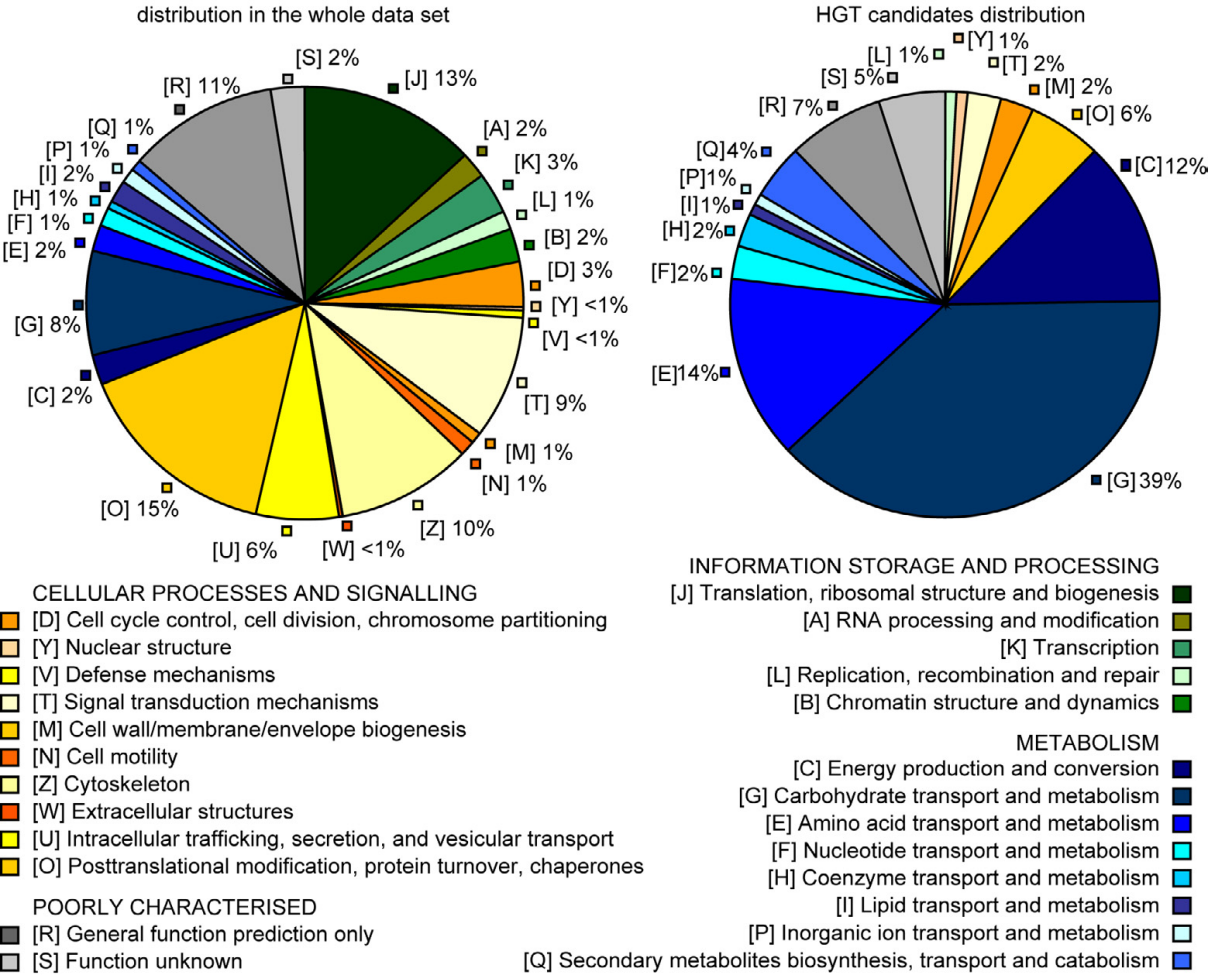
**Distribution of functional classes in HGT and in the complete set of sequences**. There is a clear over-representation of the metabolic KOG/COGs within the HGT candidates. green – the KOG/COG corresponding to the "information storage and processing"; yellow-orange – "cellular processes and signalling"; blue – "metabolism"; grey – "poorly characterised".

Out of 3563 clusters in our database, 2280 were assigned to at least one KOG or COG. Among the HGT candidates there is an over representation of genes involved in metabolism: while in the complete EST dataset the functions involved in Cellular process and signalling (47.0%) are prevalent, most of the HGT candidates are involved in Metabolism (75.4%) (See Figure 2).(Note that this number is an underestimate as it does not include 15 of the 30 sequences which do not belong to a KOG/COG – among which are eight xylanases, two cellulases, three pectate lyases, one uridine kinase and one α-glucosidase). Comparing the numbers of ESTs per cluster we found no indication that horizontally transferred genes are higher expressed than non-transferred ones (data not shown). 125 sequences out of the 148 HGT candidates encode enzymes (~84%), more than 35% of them are glycosyl hydrolases (GH), enzymes that are involved in the degradation of carbohydrates from the plant cell wall.

More specifically, most of the horizontally transferred enzymes are involved in the catabolism rather than in the anabolism. A description of these 148 candidates can be found in Additional file 2. Notably, we find a spectrum of enzymes in the Entodiniomorphids that is very different from that we find in the Vestibuliferids. The Vestibuliferids expressed some enolases, fructokinases, glucokinases that we did not find in the Entodiniomorphids. In contrast, the Entodiniomorphids expressed enzymes such as cellobiose phosphorylases, cellulases, xylanases, pectate lyase, aspartate-ammonia lyase and nitroreductase that were not found in the Vestibuliferid sequences. Moreover the only GHs that were found in the Vestibuliferids (lysozyme, β-hexoaminidase, 6-phospho β-glucosidase) are not fibrolytic enzymes. This matches the observation of Williams and Coleman (1992) that Vestibuliferids do not ingest fibres. These Ciliates depend on the uptake of starch and Bacteria.

### Glycosyl hydrolases (GH) and other enzymes involved in the degradation of complex carbohydrates

The HGT candidates are dominated by the presence of one class of enzymes, namely the glycosyl hydrolases. These are a widespread group of enzymes that hydrolyse the glycosidic bonds between carbohydrates, or between a carbohydrate and a non-carbohydrate moiety. Below, we described these enzymes, and more specifically the xylanases and cellulases.

We found twelve xylanases within the 148 HGT candidates. All of them are restricted to the Entodiniomorphids [*P. multivesiculatum *(2), *Epi. ecaudatum *(3), *Eud. maggii *(2), *Dip. affine *(1) and *M. medium *(4)]. Five of them are homologous to three genes of *C. acetobutylicum *(possible xylan degradation enzymes Q97TI1, Q97TI3 and Q97TI6) that are located in a single operon indicating that there might have been only a single transfer event involving a piece of DNA containing the whole operon. In this case, we may expect that the other homologues of the xylanase genes that were located on the same operon in *C. acetobutylicum *but that were not present in our set, are present in *M. medium*, *P. multivesiculatum *and *E. maggii *genomes. On the other hand, without complete genome data, we cannot exclude gene loss either. Table 2 describes domains present in these xylanases genes found in Ciliates, and the domains found in the other glycosyl hydrolases.

The set of HGT candidates also contains nine cellulases, which were only found in the Entodiniomorphids (Epi. ecaudatum (7) and P. multivesiculatum (2)). Seven of them belong to GH family 5 and only contain a cellulase domain. AM053298 belongs to GH family 9 and contains a glycol_hydro_9 domain. AM055058 contains an endoglucanase E like domain. Some cellulolytic Bacteria and rumen Fungi have cellulosome, which is a multi-enzyme complex for the degradation of plant cell wall polysaccharides [22]. This cellulosome consists of a scaffoldin protein that binds several glycosyl hydrolases via specialized intermodular "cohesin-dockerin" interactions. Finding such protein could reinforce the prokaryotic origin of the cellulases. However, we found no evidence for the presence of a scaffoldin and any cohesin domain was detected in the ciliate sequences. This could be explained by the fact that the occurrence of cellulases was restricted to those species that are able to ingest plant fibres. Therefore, it is likely that these cellulases do not require cohesin domains as is the case in bacterial or fungal cellulases, which are excreted into the environment. Our results on expressed genes are consistent with previous work on enzyme activities. We found cellulases in *Epidinium *confirming previous work using recombinant DNA technology [14], in which an *Epidinium *cellulase was shown to be functional. Furthermore, the role of *P. multivesiculatum *in cellulose digestion [23] is consistent with the presence of a cellulase in our *Polyplastron *sequences. Conversely, the absence of cellulase genes among the ESTs of *Entodinium*, is consistent with *in vitro *studies of *Entodinium *in which no cellulase activity was detected [24,25].

Beside the xylanases and cellulases mentioned above, we also found other enzymes involved in the degradation of the complex carbohydrates: an α-glucanotransferase, a β-hexosaminidase, a 6-phospho-β-glucosidase, three pectate lyases, a pectin degradation protein, four cellobiose phosphorylases, two glycosidases, an α-xylosidase, an α-glucosidase, a chitinase, a lysozyme, a polygalacturonase, a carbohydrate esterase, a sorbitol dehydrogenase and a galactoside O-acetyltransferase, two fructokinases, an aldose epimerase precusrsor.

### Adaptation to an anaerobic environment

Among the other HGT candidates are an Fe-hydrogenase and a malic enzyme, which are of interest because of their link with hydrogenosomes, membrane-bound organelles present in a number of anaerobic organisms such as the ciliate *Nyctotherus ovalis *[26], the chytrid fungi *Neocallimastix *and *Piromyces*, and the Parabasalian flagellate *Trichomonas vaginalis*. Most hydrogenases are found in such membrane-bound organelles, but there are exceptions for example in *Giardia *and *Entamoeba *where hydrogenases are located in the cytoplasm [27,28]. Similarly, also malic enzymes are found in various compartments of the eukaryotic cell: in *Trichomonas vaginalis*, a cytosolic malic enzyme of bacterial origin was found as well as a hydrogenosomal homologue [29]. Until now hydrogenosomes of rumen ciliates have not been studied in more detail, and it remains unclear as to whether all rumen Ciliates possess hydrogenosomes [30].

Another enzyme, Pyrophosphate-fructose 6-phosphate 1-phosphotransferase (EC: 2.7.1.90) can also be linked to adaptation to an anaerobic environment. This enzyme catalyzes the phosphorylation of D-fructose 6-phosphate to D-fructose 1,6 biphosphate, using PPi rather than the standard 6-phosphofructokinase (EC: 2.7.1.11) which uses ATP. PPi dependent phosphofructokinase typically occurs, besides in plants, among anaerobic unicellular species, and has been suggested to be an adaptation to a situation where the glycolysis is the sole source of ATP [31-33].

In the HGT candidates we also found some enzymes involved in nitrogen metabolism: four nitroreductases, two aspartate-ammonia ligases (EC: 6.3.1.1), an aspartate-ammonia lyase (4.3.1.1) and a NAD specific glutamate dehydrogenase (EC: 1.4.1.3). These last three enzymes catalyse the reversible reactions between ammonia and the respective amino-acids: L-Asparagine, L-Aspartate and L-Glutamate. In the rumen, the proteins are degraded into amino-acids, which are then rapidly deaminated to form ammonia. Ammonia can then be excreted via urine and faeces or, alternatively assimilated by ruminal Bacteria [34] or by the Ciliates themselves. Indeed a glutamate dehydrogenase (GDH) probably acquired through HGT, has already been described in *Entodinium caudatum*. This GDH was cloned and expressed in *E. coli*, where it expressed a high affinity for ammonia and α-ketoglutarate and a low affinity for glutamate, suggesting that the enzyme may be involved in the assimilation of ammonia by *Ent. caudatum *in the rumen [15].

## Discussion

### Detecting HGT by a phylogenetic approach after selecting candidates by Best Hit

A number of methods have been proposed to detect horizontal gene transfer, varying from Best Hit approaches [35] to phylogenetic methods [36] and methods that examine aberrant codon usage [37]. Here we have combined a fast screening "Best Hit" method for the detection of potential candidates followed by two levels of phylogenetic analyses. First, we used the Best Hit method to restrict the data set of potential candidates. Then, the fast Neighbor-Joining method highlighted the sequences that clustered within the Bacteria. In the final step we used a Maximum Likelihood approach which is the more time consuming, but also a more reliable phylogenetic method. Such a multi-step approach appears well suited for the detection of likely candidates when doing Maximum Likelihood analyses for all genes would be too time consuming. The large number of phylogenetic trees that had to be examined prompted us to use an automated method for their analysis: the examination of the species composition of the second smallest partition, allowing us to pinpoint the Firmicutes as the largest group of potential donors of genes.

Our large-scale analysis allowed us nevertheless to identify the few genes previously shown from small scale analyses, to have arisen through HGT (cellulase [14], xylanase [7,15]), thereby confirming the usefulness and validity of our method.

### HGT frequency

HGT seems to be an important process in the evolution of rumen Ciliates. In total 4.1% of the ESTs available for these ciliates appear to have been acquired by HGT. This number is at least one order of magnitude higher than the level of HGT found in (plant-pathogenic) nematodes (16 in the whole genome, i.e. 16 in roughly 19,000 genes, less than 0.1%) [8] and about four times the 1% of potential HGT's found in (the parasite) *Entamoeba histolytica *[9], although these differences might be biased due to the usage of EST data instead of DNA based gene predictions. However, even under the (unrealistic) assumption that the 148 candidates genes for HGT in rumen ciliates described here represent the vast majority of all HGT genes in the genome of a "standard" rumen ciliate with more than 30,000 genes, the incidence of HGT would still be about 0.5%.

The low number of transfers in nematodes (and multicellular eukaryotes in general) might be due to the fact the transferred genes must be incorporated into the germ line to be inherited. This hampers, for principal reasons, HGT into the genome of multicellular organisms. In contrast, *Entamoeba histolytica*, which exhibits a genome-wide HGT incidence of about 1%, is a unicellular eukaryote without any germ-line soma differentiation. Ciliates are in an intermediate position between unicellular organisms and multicellular organisms, being unicellular organisms, but possessing a differentiated germ-line (the micronucleus), and a somatic nucleus (the macronucleus), which accounts for all transcriptional activities during the vegetative growth of the cell. Therefore, the high incidence of evolutionary HGT might be surprising in the first instance. However, the unusually high incidence of HGT in ciliates can be explained by (i) their bacteriovory, which eventually leads to the release of bacterial DNA into the cytoplasm, and (ii) their nuclear dimorphism, which allows amplification of foreign DNA in the macronucleus [38]. Furthermore, Ciliates possess a sophisticated machinery of enzymes facilitating extended genome reorganizations obviously allowing the assembly of DNA into novel genetic environments [39].

The level of HGT in Ciliates seems more comparable to that observed in Bacteria (~10%) [37]. Most of the studies about HGT within Bacteria are based on the nucleotide composition as it has been postulated that transferred genes retain the nucleotide composition of the donor for some time [40]. This is however a relatively poor indicator[41], particularly in the case of genes which have been incorporated long ago.

### Adaptive value of HGT in exploitation of new niches

The vast majority of the transferred genes that we found in this study are involved in the degradation of plant cell wall derived carbohydrates, indicating an adaptation to the carbohydrate-rich environment in which these Ciliates live.

Interestingly, also the few other examples of HGT from Bacteria to Eukaryotes tend to involve complex carbohydrate degradation enzymes. Previously documented examples include an endo-1,4-mannanase (a glycosyl hydrolase) in *Piromyces *[42], a β-1,4-endoglucanase (cellulase) [8,43], an endoglucanase and probably other glycoside hydrolases in *Orpinomyces*, a rumen fungus [6] and pectinases in the Nematodes [8]. Here we have shown that carbohydrate degrading enzymes also dominate in a large set of horizontally transferred genes. In contrast, in the analysis of horizontal gene transfer between e.g. Archaea and Bacteria, the transferred functions are not specifically involved in the breakdown of complex carbohydrates although there is a dominance of "operational" proteins over "informational" proteins [44]. The HGT candidates we found are mainly encoding proteins involved in the degradation of complex plant cell wall derived carbohydrates reinforcing the interpretation that genes were only retained that provide an evolutionary advantage for the host in the particular ecological niche. Thus, we were able to substantiate our postulate that the lifestyle of ciliates in the gastro-intestinal tract of animals and their unique genome organisation, i.e. the presence of a macronucleus with highly processed, gene-dense chromosomes, together with a germ-line nucleus that tolerates large genome rearrangements [39], would provide favourable circumstances for the acquisition of foreign DNA. The observed high rate of HGT makes rumen Ciliates ideal bio-monitors for identification of potential gene transfer events in the gastro-intestinal tract of farm animals. The frequency of the historical transfers observed here approaches the levels of bacterial HGTs. As Ciliates ingest Bacteria, it also supports the "You are what you eat" hypothesis, since the sources for the historical HGT were clearly Bacteria which are related to Bacteria which are abundant in the rumen and gut of herbivorous mammals.

## Conclusion

We have screened a large set of ESTs from Rumen Ciliates, Eukaryotes that live in the foregut of the ruminant, by combining two methods: Best Hit and phylogenetic analyses. The amount of HGT (4.1%) is larger than what has ever been described in Eukaryotes. The results are consistent in the sense that the Horizontal Transfers are dominated by genes that internally encode enzymes involved into a few, specialized metabolic processes. Rumen Ciliate species live an anaerobic enviroment that is characterized by the availability of a rich source of plant polymers. Since Ciliates *per se *(c.f. the *Tetrahymena *genome) seem to possess only a limited repertoire of enzymes allowing the catabolism of such carbohydrates, a substantial fraction of the genes involved in the breakdown of carbohydrates in the rumen Ciliates appears to be acquired from Firmicutes and other Bacteria, which are well represented in the rumen.

Thus the horizontally transferred genes identified in this research may have greatly facilitated the Ciliates' colonization of their rumen habitat.

## Methods

### Construction of a cDNA library and sequencing

cDNA libraries were constructed from the Ciliates *Ent. simplex*, *Ent. caudatum*, *Eud. maggii*, *M. medium*, *Dip. affine*, *P. multivesiculatum *and *Epi. ecaudatum *(all Entodiniomorphids) and the Vestibuliferids *I. prostoma*, *I. intestinalis *and *Das. ruminantium *as described previously [45] and cloned into λ-Zap (Stratagene) following the manufacturer's instructions. Randomly picked clones were selected from *P. multivesiculatum *(721), *Epid. ecaudatum *(681), *Eud. maggii *(638), *Ent. caudatum *(1207), *I. prostoma *(638), and *Das. ruminantium *(595) for sequencing. In addition 96 clones were selected randomly from each of the other libraries for sequencing. The ciliates were maintained as well-defined monocultures in fistulated sheep.

DNA sequencing was performed on sequencers purchased from either PE Applied Biosystems, Beckman Coultard or Shimazu (PE Applied Biosystems ABI 373A and 377, a Genetic Analyzer – CEQ 8000 by Beckman).

### ESTs sequences

4768 ESTs (Expressed Sequence Tags) of rumen Ciliates were sequenced from 10 species of protozoa. Vectors and linkers were automatically filtered out from the data. First the vector pieces were detected using a Blastn search against the pBKCMV vector sequence on a Paracel genematcher machine using the same parameters as VecScreen (-q -5 -G 3 -E 3 -e 700 -Y 1.75e12 -F mD) [46].

Regions showing similarity to the pBKCMV vector were trimmed from the ends of the sequences. If there was remaining vector in the middle, it was removed and the longer of the two remaining fragments was kept. Subsequently, sequences that had less than 100 bases after trimming were discarded. After this filtering procedure, the dataset contained 4335 sequences.

Furthermore, the sequences were analyzed for contamination from other organisms by aligning against the EMBL database using Megablast [47] (version 2.2.4 [Aug-26-2002]) (identity ≥94% and a match length ≥40% of the sequence). This procedure removed 11 contaminated sequences (mainly by *Escherichia coli*). The remaining 4324 sequences varied in size between 100 and 896 nucleotides (nt), with an average length of 527 nt. By homology with known proteins we could get an estimate of the percentage of the genes covered by the Ciliate ESTs. The homology covered from 3% to 100% of the length of a known gene in another species, with a mean value of 57%. The 4324 sequences are distributed as follows: 715 sequences from *P. multivesiculatum*, 595 from *Epi. ecaudatum*, 543 from *Eud. maggii*, 1062 from *Ent. caudatum*, 27 from *Ent. simplex*, 10 from *Dip. affine*,151 from *M. medium*, 546 from *I. prostoma*, 84 from *I. intestinalis *and 591 from *Das. ruminantium*.

### Data storage and clustering

The sequences and the results of the analyses performed were stored in a relational MySQL database. In order to minimize the redundancy of the data, we aligned the dataset to itself using SWN (Smith-Waterman (SW) comparison at the nucleotide level). Sequences pairs having an identity ≥97% over at least 100 bp were considered to be from the same gene and were put in the same cluster. Sequences sharing less or no sequence similarity can be in the same cluster as long as they are linked via other sequences.

### Function assignment

Function assignment of the sequences was performed on three levels:

1) The protein domain structure was predicted by comparing the Hidden Markov Models (HMM) from PFAM (Protein Families Database) [48] with our dataset, using an e-value threshold of 0.01. Analysis of the O-Glycosides hydrolases was further performed with the CDD (Conserved Domain Database)[49] and SMART (Simple Modular Architecture Research Tool) [50].

2) The cDNAs were assigned to COGs (Clusters of Orthologous Groups of genes, [51]) based on sequence similarity to existing COGs in a Cognitor-like procedure [52]. The COG assignment yields a higher resolution prediction of the molecular function than a gene family assignment derived from PFAM. In addition, the COGs provide a classification of cellular roles for all the molecular protein functions that we used to classify the EST data. The COG database contains a set of all proteins and protein fragments (for fusion proteins) from a set of 66 complete unicellular genomes (COG) and a set of 7 complete eukaryotic genomes (KOG). All proteins are compared to the COG and KOG sets using SWX (SW comparison of DNA translated in the six reading frames against a protein database). We use a conservative variant of the Cognitor to match cDNAs to a COG: when the three first significant (e < 0.01) hits of the query sequence match the same COG, then this COG is assigned to that region of the query sequence. In the results we used the notation "KOG/COG" because we used the information from both sets. We first examine the KOG (eukaryotic set); in cases where no KOG was attributed to the EST sequence, the COG information was used.

3) Sequences were compared to version 42.8 of the Swissprot database (January 2004) to annotate each sequence according to a restricted vocabulary. This comparison was performed with SWX. Sequences with significant matches (e-value < 1.10–5) were annotated according to their most significant hit.

In addition, the CAZy database which contains the description of enzymes degrading, modifying, or creating glycosidic bonds[53], and the KEGG database which contains a description of enzymes and their pathway [54] were used in the analysis.

All sequence comparisons were run on a Paracel Genematcher using the Smith-Waterman [55] algorithm for pairwise sequence comparisons and Hidden Markov Models for sequence-to-profile comparisons. The output was subsequently analyzed in a Linux environment using Perl scripts.

### Best Hit

We performed a SWX search against all complete proteomes (see below; e-value < 0.01) to find in which species we encountered the most similar sequence (Best Hit).

### Including other ciliate genomes

Within the set of 148 predicted proteomes, *Plasmodium falciparum *(Apicomplexa), is evolutionarily the closest organism to the Ciliates. In order to distinguish events that could have occurred in the Ciliates before their colonization of the rumen environment from more recent events in the rumen we added the nucleotide data from the non-rumen ciliate *Tetrahymena thermophila *(*Tetrahymena *Genome Database) to our analyses.

First a tSWX (a SW search with translated nucleotides against a translated nucleotides database) comparison against the *T. thermophila *genome was performed. (nov. 2003, TIGR; E-value threshold 1.10–5, match length > 30 amino acids). The *T. thermophila *matching sequences were retrieved and translated in the 6 frames. The frame that matched the rumen ciliate was included in the tree (see below).

### Phylogenetic analyses

To derive a phylogenetic tree for each cluster of ESTs we used the following procedure: First the longest sequence of the cluster was translated into six reading frames and used as a query to search the published genomes. Homologous sequences were retrieved using SWP (SW at the protein level) with an e-value threshold of 1.10–5, *T. thermophila *homologs were added when available, and the EST was aligned with its homologs using ClustalW [56]. Subsequently phylogenetic trees were constructed using Neighbor-Joining as implemented in ClustalW (excluding positions with gaps and using 1000 bootstraps). The multiple alignments are based on the Ciliate EST sequences compared with the full length sequences of the other organisms. Trees are constructed solely based on the homologous positions after selecting the conserved positions and removing the gaps from the alignment The minimal number of conserved positions used to construct the tree is 58 amino acids (except for one xylanase that was much shorter but was based a on manual check of the alignment and of the sequences present). The maximal length was 369 amino acids, the mean length was 172.5 amino acids.

**Figure 3:**
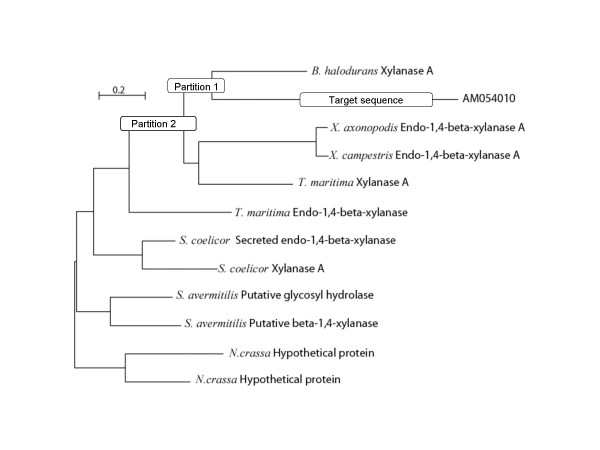
**A likely horizontally transferred gene in Entodinium**. The tree contains the target sequence (AM054010) and its homologuous proteins. To determine whether the target sequence is HGT candidate, an algorithm examines the presence or absence of Bacteria, Archaea and Eukaryotes in the second smaller partition containing the target sequence. In the example, the smallest second partition containing the target sequence contains also the sequences from *Bacillus halodurans*, *Xanthomonas axonopodis*, *Xanthomonas campestris *and *Thermotoga maritima*, which are all Bacteria. AM054010 was therefore retained as a HGT candidate.

Those ciliate sequences that clustered within a bacterial partition of the tree were considered HGT candidates. To automatically detect such cases, an algorithm examined the species composition of the "tree neighbourhood" of the ciliate sequence. By requiring that the second-smallest partition containing the ciliate sequence (Figure 3) is fully composed of bacterial sequences (plus our ciliate sequence) we assured that the ciliate sequence clustered "within" the bacterial sequences. In principle we do not know the root of the tree, and it is therefore hard to argue that when a query sequence clusters within a set of Bacterial sequences it is actually derived from a Bacterial sequence. The principle of selecting the second-smallest partition is that we assume that the root of the tree lies outside that (small) partition.

Subsequently for the selected cases we obtained high quality trees with a different methodology. We used Muscle [57] to obtain the sequence alignments, and for the positions without gaps we obtained phylogenies using Maximum Likelihood as calculated by MrBayes [58,59], using four gamma-distributed rate categories plus invariant positions and the Poisson amino acid similarity matrix. Again the "second-smallest partition" algorithm was used to obtain HGT candidates.

For the HGT candidates identified here, the annotations (See previous paragraph) were manually examined based on their phylogenetic tree, to assure that sequences were orthologous to annotated proteins, and their domain compositions.

### Proteomes

A set of 148 complete proteomes was used, which included 16 archaeal, 116 bacterial and 16 eukaryotic proteomes. (A complete list of the proteomes included can be found in Additional file 1.)

## List of abbreviations

HGT: Horizontal Gene Transfer; BH: Best Hit; FCA: SSP: Second Smallest Partition; GH: Glycosyl Hydrolase; COG: Cluster of Orthologous Group; SW: Smith Waterman; ESTs: Expressed Sequence Tags.

## Authors' contributions

GR and MAH designed the bioinformatic experiment.

GR performed computational sequence analysis and wrote the manuscript.

JHPH and MAH initiated and coordinated the study.

TM, JPJ, DM and EN established ciliate cell lines for cDNA librairies construction.

CJN and NRMcE coordinated and participated in the generation of the cDNA libraries and the production of the EST sequences.

NN and NAT participated in the growth of clones for sequencing.

FMMcI created cDNA libraries.

AT and MM generated financial support for the sequencing of clones. KU and TN sequenced the clones.

GR, BED, NRMcE, TM, MAH, JHPH, JPJ participated in drafting the manuscript.

All authors read and approved the final manuscript.

## Supplementary Material

Additional File 1 **Distribution of the complete proteomes over the various taxa**. Eukaryotic proteomes are: Plasmodium falciparum, Guillardia theta, Candida albicans, Encephalitozoon cuniculi, Neurospora crassa, Saccharomyces cerevisiae, Schizosaccharomyces pombe, Anopheles gambiae, Caenorhabditis elegans, Drosophila melanogaster, Danio rerio (zebrafish), Fugu rubripes, Homo sapiens, Mus musculus, Rattus norvegicus, and Arabidopsis thaliana. Click here for file

Additional File 2 **Classification of the 148 HGT candidates**. Annotation was given on the basis of the complete analysis (homology with known proteins, homology to proteins of a KOG/COG, PFAM domains and confirmed from the tree). CCD: Complex carbohydrates degradation, F: Fermentation, G: Glycolysis, PD: protein degradation, NM: Nitrogen metabolism. Composition of the Smith Waterman comparison results (Hit; E-value < 1·10–5) or second-smallest partition (SSP): B: only Bacteria; AB: Bacteria and Archaea; A: only Archaea. Click here for file

## Figures and Tables

**Table 1:** Species distribution of the ciliate dataset. Indicated are the total number of sequences per species and the number of clusters that were obtained after combining the sequences likely derived from a same gene. 377 clusters have a size varying between 2 and 21 sequences, the rest of the clusters are singletons.

**Organism**	**Number of sequences**	**Number of clusters**	**number of clusters ≥2 sequences** ** (size maximum of the cluster)**	**number of clusters singletons**
*P. multivesiculatum*	*715*	*502*	*115 (21)*	*387*
*Epid. ecaudatum*	*595*	*530*	*46 (5)*	*484*
*Eud. maggii*	543	500	32 (4)	468
*I. prostoma*	546	442	43 (12)	399
*I. intestinalis*	84	81	2 (3)	79
*Das. ruminantium*	591	421	61 (20)	360
*Ent. caudatum*	1062	901	76 (18)	825
*Ent. simplex*	27	27	0	27
*Dip. affine*	10	10	0	10
*M. medium*	151	149	2 (2)	147

TOTAL	4324	3563	377	3186

**Table 2:** Enzymes involved in the degradation of complex carbohydrates that appear to have been acquired by HGT.

Organism	Accession number	Annotation	Domains
*Das. ruminantium*	AM051680	6-phospho-beta-glucosidase	glycosyl hydrolase family 1
*P. multivesiculatum*	AM055068	Aldose 1-epimerase precursor	aldose 1 epimerase
*Epi. ecaudatum*	AM053198	alpha glucanotransferase	4-alpha-glucanotransferase
*P. multivesiculatum*	AM055071	alpha-glucosidase	no
*P. multivesiculatum*	AM055169	Alpha-xylosidase	Glyco_hydro_31
*Iso. prostoma*	AM054399	Beta-hexosaminidase	glycosyl hydrolase family 3, N terminal domain
*Epi. ecaudatum*	AM053419	carbohydrate esterase	rhamnogalaturan acetyltransferase
*Epi. ecaudatum*	AM053258	Cellobiose phosphorylase	CBM_X
*Epi. ecaudatum*	AM053258	Cellobiose phosphorylase	Glyco_transf_36
*Eud. maggii*	AM053819	Cellobiose phosphorylase	CBM_X
*Eud. maggii*	AM053819	Cellobiose phosphorylase	Glyco_transf_36
*Eud. maggii*	AM053820	Cellobiose phosphorylase	no
*P. multivesiculatum*	AM055116	Cellobiose phosphorylase	no
*Epi. ecaudatum*	AM053290	cellulase	Glyco_hydro_5
*Epi. ecaudatum*	AM053291	cellulase	Glyco_hydro_5
*Epi. ecaudatum*	AM053292	cellulase	Glyco_hydro_5
*Epi. ecaudatum*	AM053294	cellulase	Glyco_hydro_5
*Epi. ecaudatum*	AM053295	cellulase	Glyco_hydro_5
*Epi. ecaudatum*	AM053297	cellulase	Glyco_hydro_5
*Epi. ecaudatum*	AM053298	cellulase	Glyco_hydro_9
*P. multivesiculatum*	AM055058	cellulase	endoglucacanase E like
*P. multivesiculatum*	AM055119	cellulase	Glyco_hydro_5
*Epi. ecaudatum*	AM053266	Chitinase D precursor	Glyco_hydro_18
*Dip. affine*	AM052171	Endo-1,4-beta-xylanase	Glyco_hydro_10
*Epi. ecaudatum*	AM053241	Endo-1,4-beta-xylanase	Glyco_hydro_10
*M. medium*	AM055031	Endo-1,4-beta-xylanase precursor	Glyco_hydro_11
*M. medium*	AM055032	Endo-1,4-beta-xylanase precursor	Glyco_hydro_11
*Epi. ecaudatum*	AM053745	Endo-1,4-beta-xylanase T	Glyco_hydro_11
*Das. ruminantium*	AM051785	Fructokinase	ROK
*Das. ruminantium*	AM051789	Fructokinase	ROK
*Ent. caudatum*	AM052478	galactoside O-acetyltransferase	Trimeric LpxA like enzyme family
*Epi. ecaudatum*	AM053257	glycosidase	NLPC_P60
*P. multivesiculatum*	AM055193	glycosidase	chitinase_glyco_hydro_19
*Iso. prostoma*	AM054501	Lysozyme	Phage_lysozyme
*Epi. ecaudatum*	AM053368	Pectate lyase	no
*Epi. ecaudatum*	AM053374	Pectate lyase	no
*P. multivesiculatum*	AM055221	Pectate lyase	no
*M. medium*	AM054934	pectin degradation protein	no
*P. multivesiculatum*	AM055224	Polygalacturonase	Glyco_hydro_28
*Epi. ecaudatum*	AM053746	xylanase	Glyco_hydro_10
*Eud. maggii*	AM053864	xylanase	cellulose binding domain: CBD_IV
*Eud. maggii*	AM054010	xylanase	Glyco_hydro_10
*M. medium*	AM055027	xylanase	alpha-L arabinanase
*M. medium*	AM055030	xylanase	cellulose binding domain: CBD_II
*P. multivesiculatum*	AM055577	xylanase	glycol_hydro_30
*P. multivesiculatum*	AM055581	xylanase	cellulose binding domain: CBD_IV alpha-L arabinanase
