# Supplementary material for: Horizontal gene transfer from Bacteria to rumen Ciliates indicates adaptation to their anaerobic, carbohydrates-rich environment
Source: BMC Genomics. 2006 Feb 10;7:22. doi: 10.1186/1471-2164-7-22 (PMC1413528; doi:10.1186/1471-2164-7-22)
Supplement: Additional File 1 — Distribution of the complete proteomes over the various taxa. Eukaryotic proteomes are: Plasmodium falciparum, Guillardia theta, Candida albicans, Encephalitozoon cuniculi, Neurospora crassa, Saccharomyces cerevisiae, Schizosaccharomyces pombe, Anopheles gambiae, Caenorhabditis elegans, Drosophila melanogaster, Danio rerio (zebrafish), Fugu rubripes, Homo sapiens, Mus musculus, Rattus norvegicus, and Arabidopsis thaliana. [file 1471-2164-7-22-S1.doc]

**Additional file 1: Distribution of the complete proteomes over the various taxa.**

| **Class** | **Number of organisms in the proteome set** |
| --- | --- |
| Bacteria Firmicutes | 34 |
| Bacteria Proteobacteria Gammaproteobacteria | 30 |
| Bacteria Proteobacteria Alphaproteobacteria | 12 |
| Bacteria Actinobacteria | 11 |
| Archaea Euryarchaeota | 10 |
| Eukaryota Fungi/Metazoa group Metazoa | 8 |
| Bacteria Chlamydiae/Verrucomicrobia group Chlamydiae | 7 |
| Eukaryota Fungi/Metazoa group Fungi | 5 |
| Bacteria Proteobacteria delta/epsilon subdivisions | 4 |
| Bacteria Proteobacteria Betaproteobacteria | 4 |
| Bacteria Cyanobacteria | 4 |
| Bacteria Spirochaetes | 3 |
| Archaea Crenarchaeota | 3 |
| Bacteria Fusobacteria Fusobacteria | 2 |
| Eukaryota Viridiplantae | 1 |
| Eukaryota Cryptophyta Cryptomonadaceae | 1 |
| Eukaryota Alveolata Apicomplexa | 1 |
| Bacteria Thermotogae | 1 |
| Bacteria Deinococcus‑Thermus Deinococci | 1 |
| Bacteria Bacteroidetes/Chlorobi group Chlorobi | 1 |
| Bacteria Bacteroidetes/Chlorobi group | 1 |
| Bacteria Aquificae | 1 |
| Archaea Euryarchaeota Halobacteria | 1 |
| Archaea Euryarchaeota Archaeoglobi | 1 |
| Archae Crenarchaeota Thermoprotei | 1 |

Eukaryotic proteomes are: Plasmodium falciparum, Guillardia theta, Candida albicans, Encephalitozoon cuniculi, Neurospora crassa, Saccharomyces cerevisiae, Schizosaccharomyces pombe, Anopheles gambiae, Caenorhabditis elegans, Drosophila melanogaster, Danio rerio (zebrafish), Fugu rubripes, Homo sapiens, Mus musculus, Rattus norvegicus, and Arabidopsis thaliana.
