# Supplementary material for: Horizontal gene transfer from Bacteria to rumen Ciliates indicates adaptation to their anaerobic, carbohydrates-rich environment
Source: BMC Genomics. 2006 Feb 10;7:22. doi: 10.1186/1471-2164-7-22 (PMC1413528; doi:10.1186/1471-2164-7-22)
Supplement: Additional File 2 — Classification of the 148 HGT candidates. Annotation was given on the basis of the complete analysis (homology with known proteins, homology to proteins of a KOG/COG, PFAM domains and confirmed from the tree). CCD: Complex carbohydrates degradation, F: Fermentation, G: Glycolysis, PD: protein degradation, NM: Nitrogen metabolism. Composition of the Smith Waterman comparison results (Hit; E-value < 1·10–5) or second-smallest partition (SSP): B: only Bacteria; AB: Bacteria and Archaea; A: only Archaea. [file 1471-2164-7-22-S2.doc]

**Additional file 2: Classification of the 148 HGT candidates.**

Annotation was given on the basis of the complete analysis (homology with known proteins, homology to proteins of a KOG/COG, PFAM domains and confirmed from the tree). CCD: Complex carbohydrates degradation, F: Fermentation, G: Glycolysis, PD: protein degradation, NM: Nitrogen metabolism.

Composition of the Smith Waterman comparison results (Hit; E-value < 1·10^-5^) or second-smallest partition (SSP): B: only Bacteria ; AB: Bacteria and Archaea ; A: only Archaea.

| **Access number** | **Organism** | **Hit** | **SSP** | **Annotation** | **EC number** | **Function** |
| --- | --- | --- | --- | --- | --- | --- |
| AM051680 | *Dasytricha ruminantium* |  | B | 6-phospho-beta-glucosidase | 3.2.1.86 | CCD |
| AM053775 | *Eudiplodinium maggii* |  | B | Acetate kinase | 2.7.2.1 | F |
| AM054515 | *Isotricha  prostoma* |  | B | Alcohol dehydrogenase | - | F |
| AM054285 | *Isotricha intestinalis* |  | B | Aldehyde dehydrogenase | 1.2.1.3 | F |
| AM053404 | *Epidinium ecaudatum* |  | B | aldo-keto reductase | - | - |
| AM055068 | *Polyplastron multivesiculatum* |  | B | Aldose 1-epimerase precursor | 5.1.3.3 | CCD |
| AM053198 | *Epidinium ecaudatum* |  | B | alpha glucanotransferase | 2.4.1.25 | CCD |
| AM055071 | *Polyplastron multivesiculatum* | B | B | alpha-glucosidase | 3.2.1.20 | CCD |
| AM055169 | *Polyplastron multivesiculatum* |  | AB | Alpha-xylosidase | 3.2.1.- | CCD |
| AM053837 | *Eudiplodinium maggii* |  | B | aminopeptidase | 3.4.22.40 | PD |
| AM054362 | *Isotricha  prostoma* | B | B | aminotransferase | 2.6.1.37 | - |
| AM053235 | *Epidinium ecaudatum* |  | B | Aspartate ammonia-lyase | 4.3.1.1 | NM |
| AM053236 | *Epidinium ecaudatum* | B | B | Aspartate--ammonia ligase | 6.3.1.1 | NM |
| AM055094 | *Polyplastron multivesiculatum* | B | B | Aspartate--ammonia ligase | 6.3.1.1 | NM |
| AM054399 | *Isotricha  prostoma* | B | B | Beta-hexosaminidase | 3.2.1.52 | CCD |
| AM053808 | *Eudiplodinium maggii* | AB | B | Biotin ligase | 6.3.4.15 | - |
| AM054287 | *Isotricha intestinalis* |  | B | Biotin synthase | 2.8.1.6 | - |
| AM053419 | *Epidinium ecaudatum* |  | B | carbohydrate esterase | 3.1.1.- | CCD |
| AM051830 | *Dasytricha ruminantium* | B | B | cell membrane protein | - | - |
| AM053342 | *Epidinium ecaudatum* | B | B | cell surface protein | - | - |
| AM053343 | *Epidinium ecaudatum* |  | B | cell surface protein | - | - |
| AM054011 | *Eudiplodinium maggii* |  | B | cell surface protein | - | - |
| AM053912 | *Eudiplodinium maggii* | B | B | cell surface protein | - | - |
| AM052456 | *Entodinium caudatum* |  | B | cell surface protein | - | - |
| AM053344 | *Epidinium ecaudatum* | B | B | cell surface protein | - | - |
| AM053913 | *Eudiplodinium maggii* |  | B | cell surface protein | - | - |
| AM055412 | *Polyplastron multivesiculatum* | B | B | cell surface protein | - | - |
| AM055303 | *Polyplastron multivesiculatum* | B | B | cell surface protein | - | - |
| AM055259 | *Polyplastron multivesiculatum* | B | B | cell surface protein | - | - |
| AM055261 | *Polyplastron multivesiculatum* |  | B | cell surface protein | - | - |
| AM053258 | *Epidinium ecaudatum* | B | B | Cellobiose phosphorylase | 2.4.1.20 | CCD |
| AM053819 | *Eudiplodinium maggii* | B | B | Cellobiose phosphorylase | 2.4.1.20 | CCD |
| AM053820 | *Eudiplodinium maggii* |  | B | Cellobiose phosphorylase | 2.4.1.20 | CCD |
| AM055116 | *Polyplastron multivesiculatum* |  | B | Cellobiose phosphorylase | 2.4.1.20 | CCD |
| AM053297 | *Epidinium ecaudatum* | B | B | cellulase | 3.2.1.4 | CCD |
| AM053298 | *Epidinium ecaudatum* |  | B | cellulase | 3.2.1.4 | CCD |
| AM053290 | *Epidinium ecaudatum* | B | B | cellulase | 3.2.1.4 | CCD |
| AM053291 | *Epidinium ecaudatum* | B | B | cellulase | 3.2.1.4 | CCD |
| AM053292 | *Epidinium ecaudatum* | B | B | cellulase | 3.2.1.4 | CCD |
| AM053294 | *Epidinium ecaudatum* | B | B | cellulase | 3.2.1.4 | CCD |
| AM053295 | *Epidinium ecaudatum* | B | B | cellulase | 3.2.1.4 | CCD |
| AM055119 | *Polyplastron multivesiculatum* | AB | AB | cellulase | 3.2.1.4 | CCD |
| AM055058 | *Polyplastron multivesiculatum* | B | B | cellulase | 3.2.1.4 | CCD |
| AM054473 | *Isotricha  prostoma* |  | B | Chaperone protein dnaK (Heat shock | - | - |
| AM053266 | *Epidinium ecaudatum* | B | B | Chitinase D precursor | 3.2.1.14 | CCD |
| AM053475 | *Epidinium ecaudatum* | B | B | conserved protein | - | - |
| AM053587 | *Epidinium ecaudatum* | AB | B | conserved protein | - | - |
| AM053648 | *Epidinium ecaudatum* | B | B | conserved protein | - | - |
| AM052836 | *Entodinium caudatum* |  | B | conserved protein | - | - |
| AM055164 | *Polyplastron multivesiculatum* | AB | B | conserved protein | - | - |
| AM055544 | *Polyplastron multivesiculatum* | B | B | conserved protein | - | - |
| AM052328 | *Entodinium caudatum* |  | B | Cysteine synthase | 2.5.1.47 | - |
| AM053717 | *Epidinium ecaudatum* | B | B | deoxyribose-phosphate aldolase | 4.1.2.4 | - |
| AM054431 | *Isotricha  prostoma* |  | B | Deoxyribose-phosphate aldolase | 4.2.1.4 | - |
| AM055135 | *Polyplastron multivesiculatum* | B | B | dipeptidase | 3.4.-.- | PD |
| AM053848 | *Eudiplodinium maggii* |  | B | DNA polymerase III, epsilon chain | - | - |
| AM052171 | *Diploplastron affine* |  | B | Endo-1,4-beta-xylanase | 3.2.1.8 | CCD |
| AM053241 | *Epidinium ecaudatum* |  | B | Endo-1,4-beta-xylanase | 3.2.1.8 | CCD |
| AM055031 | *Metadinium medium* |  | B | Endo-1,4-beta-xylanase precursor | 3.2.1.8 | CCD |
| AM055032 | *Metadinium medium* |  | B | Endo-1,4-beta-xylanase precursor | 3.2.1.8 | CCD |
| AM053745 | *Epidinium ecaudatum* |  | B | Endo-1,4-beta-xylanase T | 3.2.1.8 | CCD |
| AM051765 | *Dasytricha ruminantium* |  | AB | Enolase | 4.2.1.11 | G |
| AM054444 | *Isotricha  prostoma* |  | B | Enolase | 4.2.1.11 | G |
| AM054445 | *Isotricha  prostoma* |  | B | Enolase | 4.2.1.11 | G |
| AM054295 | *Isotricha intestinalis* |  | AB | Enolase | 4.2.1.11 | G |
| AM054296 | *Isotricha intestinalis* |  | B | Enolase | 4.2.1.11 | G |
| AM054297 | *Isotricha intestinalis* |  | B | Enolase | 4.2.1.11 | G |
| AM053355 | *Epidinium ecaudatum* |  | B | flavin oxidoreductase | 1.5.1.30 | - |
| AM053405 | *Epidinium ecaudatum* | AB | B | flavodoxin | - | - |
| AM051785 | *Dasytricha ruminantium* |  | B | Fructokinase | 2.7.1.4 | CCD |
| AM051789 | *Dasytricha ruminantium* | B | B | Fructokinase | 2.7.1.4 | CCD |
| AM052478 | *Entodinium caudatum* |  | AB | galactoside O-acetyltransferase | 2.3.1.18 | CCD |
| AM051795 | *Dasytricha ruminantium* | B | B | glucokinase | 2.7.1.2 | G |
| AM054456 | *Isotricha  prostoma* | B | B | Glucokinase | 2.7.1.2 | G |
| AM054301 | *Isotricha intestinalis* | B | B | Glucokinase | 2.7.1.2 | G |
| AM053317 | *Epidinium ecaudatum* |  | B | glucosamine 6 phosphate deaminase | 3.5.99.6 | - |
| AM053320 | *Epidinium ecaudatum* |  | B | Glutathione peroxidase homolog. | 1.11.1.9 | - |
| AM054459 | *Isotricha  prostoma* |  | B | Glutathione peroxidase homolog. | 1.11.1.9 | - |
| AM053950 | *Eudiplodinium maggii* |  | B | Glycerol-3-phosphate cytidylyltransferase | 2.7.7.39 | - |
| AM053257 | *Epidinium ecaudatum* | B | B | glycosidase | 3.2.1.- | CCD |
| AM055193 | *Polyplastron multivesiculatum* | B | B | glycosidase | 3.2.1.- | CCD |
| AM053921 | *Eudiplodinium maggii* |  | B | Hypothetical oxidoreductase yajO | 1.-.-.- | - |
| AM053922 | *Eudiplodinium maggii* |  | B | Hypothetical oxidoreductase yajO | 1.-.-.- | - |
| AM052481 | *Entodinium caudatum* |  | B | Hypothetical oxidoreductase yajO | 1.-.-.- | - |
| AM053134 | *Entodinium caudatum* | B | B | Hypothetical oxidoreductase ybiC | 1.1.1.- | - |
| AM053195 | *Epidinium ecaudatum* |  | B | Hypothetical oxidoreductase ykwC | 1.-.-.- | - |
| AM054501 | *Isotricha  prostoma* | B | B | Lysozyme | 3.2.1.17 | CCD |
| AM053359 | *Epidinium ecaudatum* |  | B | Mannitol dehydrogenase | - | - |
| AM053520 | *Epidinium ecaudatum* | B | B | membrane protease subunit | - | - |
| AM052444 | *Eudiplodinium maggii* | B | B | membrane protease subunit | - | - |
| Eec06_H12 | *Epidinium ecaudatum* |  | B | NAD specific glutamate dehydrogenase | 1.4.1.3 | NM |
| AM051841 | *Dasytricha ruminantium* |  | AB | NADP-dependent alcohol dehydrogenase | 1.1.1.2 | F |
| AM051842 | *Dasytricha ruminantium* |  | AB | NADP-dependent alcohol dehydrogenase | 1.1.1.2 | F |
| AM053942 | *Eudiplodinium maggii* | B | B | nitroreductase | - | NM |
| AM054933 | *Metadinium medium* | B | B | nitroreductase | - | NM |
| AM055214 | *Polyplastron multivesiculatum* | B | B | nitroreductase | - | NM |
| AM055216 | *Polyplastron multivesiculatum* | B | B | nitroreductase | - | NM |
| AM051849 | *Dasytricha ruminantium* |  | B | Ornithine decarboxylase | 4.1.1.17 | - |
| AM053842 | *Eudiplodinium maggii* |  | B | oxidoreductase | - | - |
| AM053368 | *Epidinium ecaudatum* | B | B | Pectate lyase | 4.2.2.2 | CCD |
| AM053374 | *Epidinium ecaudatum* | B | B | Pectate lyase | 4.2.2.2 | CCD |
| AM055221 | *Polyplastron multivesiculatum* | B | B | Pectate lyase | 4.2.2.2 | CCD |
| AM054934 | *Metadinium medium* | AB | AB | pectin degradation protein | - | CCD |
| AM055186 | *Polyplastron multivesiculatum* | B | B | Periplasmic [Fe] hydrogenase 1 | 1.12.7.2 | - |
| AM054553 | *Isotricha  prostoma* |  | B | phosphoglycerate mutase | 5.4.2.1 | G |
| AM055224 | *Polyplastron multivesiculatum* |  | B | Polygalacturonase | 3.2.1.15 | CCD |
| AM053394 | *Epidinium ecaudatum* |  | B | Porphobilinogen deaminase | 2.5.1.61 | - |
| AM054427 | *Isotricha  prostoma* |  | B | Probable cystathionine gamma-synthase | 4.2.99.9 | - |
| AM051740 | *Dasytricha ruminantium* | B | B | Probable dipeptidase A | 3.4.-.- | PD |
| AM051741 | *Dasytricha ruminantium* | B | B | Probable dipeptidase A | 3.4.-.- | PD |
| AM055136 | *Polyplastron multivesiculatum* | B | B | Probable dipeptidase A | 3.4.-.- | PD |
| AM055137 | *Polyplastron multivesiculatum* | B | B | Probable dipeptidase A | 3.4.-.- | PD |
| AM055257 | *Polyplastron multivesiculatum* | B | B | Probable dipeptidase A | 3.4.-.- | PD |
| AM052452 | *Entodinium caudatum* |  | AB | Probable NAD-dependent malic enzyme | 1.1.1.38 | - |
| AM053973 | *Eudiplodinium maggii* |  | B | Probable reductase | 1.1.-.- | - |
| AM053193 | *Epidinium ecaudatum* |  | B | Probable reductase | 1.1.-.- | - |
| AM053806 | *Eudiplodinium maggii* | B | B | protease | - | PD |
| AM055033 | *Metadinium medium* | B | B | protease | - | PD |
| AM052526 | *Entodinium caudatum* |  | B | pyrimidine nucleoside phosphorylase | 2.4.2.2 | - |
| AM051942 | *Dasytricha ruminantium* |  | B | Pyrimidine-nucleoside phosphorylase | - | - |
| AM054309 | *Isotricha intestinalis* |  | B | Pyrophosphate-fructose 6-phosphate 1-phosphotransferase | 2.7.1.90 | - |
| AM052533 | *Entodinium caudatum* |  | B | pyruvate phosphate dikinase | 2.7.9.1 | G |
| AM051899 | *Dasytricha ruminantium* |  | B | Pyruvate,phosphate dikinase | 2.7.9.1 | G |
| AM052528 | *Eudiplodinium maggii* |  | B | Pyruvate,phosphate dikinase | 2.7.9.1 | G |
| AM052480 | *Entodinium caudatum* |  | B | Pyruvate,phosphate dikinase | 2.7.9.1 | G |
| AM052531 | *Entodinium caudatum* |  | B | Pyruvate,phosphate dikinase | 2.7.9.1 | G |
| AM052534 | *Entodinium caudatum* |  | AB | Pyruvate,phosphate dikinase | 2.7.9.1 | G |
| AM054582 | *Isotricha  prostoma* |  | B | Pyruvate,phosphate dikinase | 2.7.9.1 | G |
| AM053981 | *Eudiplodinium maggii* |  | A | regucalcin | - | - |
| AM055299 | *Polyplastron multivesiculatum* | B | B | Ribose 5-phosphate isomerase | 5.3.1.6 | - |
| AM053448 | *Epidinium ecaudatum* |  | B | sorbitol dehydrogenase | 1.1.1.14 | - |
| AM053238 | *Epidinium ecaudatum* | B | B | S-ribosylhomocysteinase | 3.13.1.- | - |
| AM055300 | *Polyplastron multivesiculatum* |  | B | sugar phospate isomerase | 5.3.1.6 | - |
| AM052591 | *Entodinium caudatum* |  | AB | thioredoxin | 1.8.1.9 | - |
| AM051940 | *Dasytricha ruminantium* |  | AB | Thioredoxin 2 (DmTrx-2). | 1.8.1.9 | - |
| AM053467 | *Epidinium ecaudatum* |  | B | UDP-glucose 4-epimerase | 5.1.3.2 | - |
| AM054635 | *Isotricha  prostoma* |  | B | UDP-glucose 4-epimerase | 5.1.3.2 | - |
| AM055325 | *Polyplastron multivesiculatum* |  | A | UDP-glucose 6-dehydrogenase | 1.1.1.22 | - |
| AM053453 | *Epidinium ecaudatum* | B | B | uridine kinase | 2.7.1.48 | - |
| AM053746 | *Epidinium ecaudatum* |  | B | xylanase | 3.2.1.8 | CCD |
| AM054010 | *Eudiplodinium maggii* |  | B | xylanase | 3.2.1.8 | CCD |
| AM053864 | *Eudiplodinium maggii* | B | B | xylanase | 3.2.1.8 | CCD |
| AM055030 | *Metadinium medium* | B | B | xylanase | 3.2.1.8 | CCD |
| AM055027 | *Metadinium medium* | B | B | xylanase | 3.2.1.8 | CCD |
| AM055577 | *Polyplastron multivesiculatum* | B | B | xylanase | 3.2.1.8 | CCD |
| AM055581 | *Polyplastron multivesiculatum* | B | B | xylanase | 3.2.1.8 | CCD |
| AM053656 | *Epidinium ecaudatum* | B | B | zeta toxin | - | - |
| AM053753 | *Epidinium ecaudatum* |  | AB | zinc-type alcohol dehydrogenase | 1.1.-.- | F |
